# Towards functional characterization of archaeal genomic dark matter

**DOI:** 10.1042/BST20180560

**Published:** 2019-02-01

**Authors:** Kira S. Makarova, Yuri I. Wolf, Eugene V. Koonin

**Affiliations:** National Center for Biotechnology Information, National Library of Medicine, Bethesda, MD 20894, U.S.A.

**Keywords:** archaeal proteins, computational biology, functional genomics

## Abstract

A substantial fraction of archaeal genes, from ∼30% to as much as 80%, encode ‘hypothetical' proteins or genomic ‘dark matter'. Archaeal genomes typically contain a higher fraction of dark matter compared with bacterial genomes, primarily, because isolation and cultivation of most archaea in the laboratory, and accordingly, experimental characterization of archaeal genes, are difficult. In the present study, we present quantitative characteristics of the archaeal genomic dark matter and discuss comparative genomic approaches for functional prediction for ‘hypothetical' proteins. We propose a list of top priority candidates for experimental characterization with a broad distribution among archaea and those that are characteristic of poorly studied major archaeal groups such as Thaumarchaea, DPANN (Diapherotrites, Parvarchaeota, Aenigmarchaeota, Nanoarchaeota and Nanohaloarchaeota) and Asgard.

## Introduction

The drop of sequencing costs over the last decade has led to a dramatic increase in the influx of new genomes into public databases. Furthermore, unlike the preceding years, most of these new genomic sequences are coming from metagenomic projects and belong to unculturable species [1,2]. In particular, metagenomics has yielded more than 10 major new archaeal groups including most of the lineages in the DPANN (Diapherotrites, Parvarchaeota, Aenigmarchaeota, Nanoarchaeota and Nanohaloarchaeota) superphylum that include, mostly, unculturable archaea with small genomes, many if not most of them, symbionts or parasites of other archaea. Affiliated with the TACK (Thaumarchaea, Aigarchaea, Crenarchaea, Korarchaea) superphylum is the Asgard group that currently consists exclusively of uncultured organisms of the putative phyla Loki-, Thor-, Odin- and Heimdallarchaeota lineages [3] that appear to be the closest archaeal relatives of eukaryotes. Additionally, new putative phyla have been discovered within the TACK superphylum including Bathyarchaeota, Geoarchaeota and Verstraetearchaeota, as well as among Euryarchaeota (Altiarchaea, Thalassoarchaea, Theionarchaea, Methanonatronarchaeia, Hadesarchaeota, Methanofastidiosa) [2,4].

For the uncultured archaea (and bacteria), gene annotation, based on comparison of protein sequences and operon organizations, is the only available source of information. Most often, the genome annotation that is deposited in public databases, such as GenBank, is generated automatically and thus is notably error-prone. Typical genome annotation errors include inaccurate gene calling whereby small ORFs (open reading frames) are falsely predicted as protein-coding genes, prediction of genes in the wrong DNA strand, prediction of ORFs in sequences that are actually non-coding, such as CRISPR arrays, and most often, erroneous assignment of start codons [5]. These annotation errors commingle with multiple gene fragments and pseudogenes that emerge through natural processes of gene degeneration. Arguably, most important, the gene annotation pipelines have to operate in a ‘safe mode', to minimize the rate of false-positive assignments. As a result, numerous sequences in the ‘twilight zone' of sequence similarity are annotated as ‘hypothetical proteins', a problem that affects, primarily, fast evolving genes, in particular, those involved in anti-parasite defense and genes that encode small proteins [6]. The quality of the annotation depends not only on the quality of the computational analyses themselves but also on the speed and completeness of the integration of new experimental data on protein functions integrated into annotation pipelines. At least in the case of archaea, annotation in public databases often runs behind experimental studies. For example, archaea-specific ribosomal proteins L45 and L47, experimentally identified in 2011 [7] and pre-rRNA processing and ribosome biogenesis proteins of the NOL1/NOP2/fmu family characterized in 1998 [8], are still not included in the annotation pipelines, so that most of the respective proteins remain ‘hypothetical'. The situation becomes even worse when it comes to the numerous confident predictions of protein functions that come from *in silico* analyses and cover several hundred protein families and several thousand ‘hypothetical' proteins. Among these predictions, there are several conserved families of membrane proteins [9], numerous genes linked to type IV pili systems [10], genes associated with various signal transduction pathways [11,12], polymorphic toxin systems [13], as well as integrated viruses and plasmids [14,15].

The mostly technical issues outlined above appear to stand behind the highest fractions of ‘dark matter' in microbial genomes. When more sensitive methods for sequence comparison and manual curation are employed, the ‘dark matter' fraction can be brought down to ∼20%, on average [16]. However, even after these substantial improvements in gene annotation, the dark matter includes millions of seemingly unique, completely uncharacterized proteins. Obviously, this number will grow fast as more genomes are sequenced, even if the fraction of dark matter in genomes remains constant or slowly drops.

An intriguing question of obvious importance is: what are the functions of these enigmatic genes? Comparative genomics and in-depth sequence analysis remain major approaches for prediction of protein functions, and the importance of this analysis further increases with the rising contribution of uncultured organisms to the genomic databases [17–19]. The growing volume and diversity of sequence databases is both a challenge — because of the increasing computational costs — and a boon to functional annotation of genes because the sensitivity of sequence searches can be dramatically increased thanks to the use of protein family profiles as queries. In addition to the increased sensitivity of sequence similarity detection, functional prediction strongly benefits also from comparative analysis of genomic contexts in increasingly diverse microbial genomes [17,20,21]. To reflect this, the COMBREX database for documenting experimental and *in silico* evidence for protein function predictions and for the prioritization of uncharacterized proteins for experimental testing has been developed [22]. Furthermore, experimental platforms for systematic validation of functional predictions produced by computational genomics have been launched, including characterization of new enzymes [23,24] and defense systems [25].

Several years ago, we undertook an initial analysis of dark matter islands in 168 archaeal genomes [26]. We found that these islands comprised ∼20% of archaeal genomes and that in-depth analysis allowed us to predict at least a general function for many of such loci and individual genes. In particular, it has been found that dark matter islands are enriched in integrated elements, novel defense systems and other genes implicated in interspecies conflicts [26]. Here, we report an analysis of the dark matter in 524 archaeal genomes covering all major archaeal lineages.

## Quantitative characteristics of the archaeal dark matter

**Figure 1.**
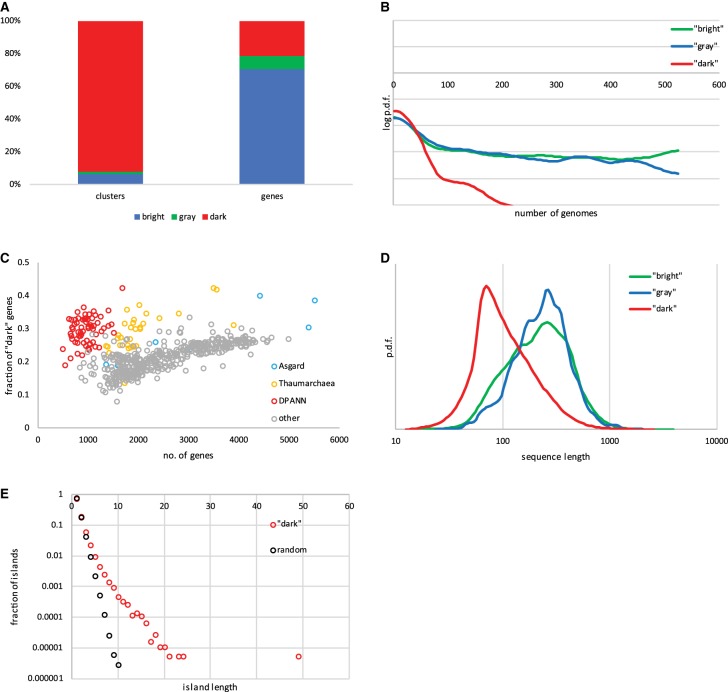
Dark matter in archaeal genomes. Amino acid sequences of proteins, encoded in 524 (nearly) completely sequenced archaeal genomes were, when possible, assigned to 13 443 arCOGs [16] and the rest were clustered together. The combination of arCOGs and clusters is referred to as ‘gene families' here and elsewhere in the text. (**A**) The relative frequencies of ‘dark' (no functional annotation), ‘gray' (general functional prediction only), and ‘bright' (functionally annotated) matter among archaeal gene families (arCOGs and clusters) and individual genes. (**B**) Distribution of the number of genomes represented in the ‘dark', ‘gray', and ‘bright matter’ gene families. The plot shows the Gaussian kernel smoothed probability density functions in log scale; the number of genomes ranges from 1 (ORFan gene) to 524 (strictly ubiquitous family). (**C**) The fraction of ‘dark matter' genes in 524 archaeal genomes. (**D**) Distribution of the sequence lengths among the ‘dark’, ‘gray', and ‘bright matter' protein families. The plot shows the Gaussian kernel smoothed probability density functions, calculated for the family consensus sequences. (**E**) Distribution of the island lengths (lengths of contiguous blocks of genes) for the ‘dark matter' genes and for a randomly selected gene set of the same size (285 155 genes).

In the archaeal genomes from the GenBank version of March 2018, the fraction of dark matter genes (those annotated as ‘hypothetical' or ‘uncharacterized’ proteins) lies with the broad 30–80% range. The database of Archaeal Clusters of Orthologous Genes (arCOGs) has been employed to assign more annotations to archaeal genes [16]. When the arCOGs are used to annotate this set of genomes, the dark matter fraction (genes that do not belong to arCOGs or belong to the uncharacterized arCOGs of the functional category S) falls to 15–40% (Supplementary Table S1). Additionally, 8% of the genes assigned to arCOGs represent a ‘gray matter', i.e. arCOGs with a general function prediction only (functional category R, Figure 1A). Overall, the dark matter dominates the diversity of the gene families (92%) but represents a minority of genes (22%; Figure 1A). This difference stems from the fact that the dark matter families are typically small and are represented in only a few genomes (Figure 1B). Only 0.1% of the dark matter families are present in over 200 (out of the 524) genomes; for comparison, 22% of the functionally annotated arCOGs cross this threshold.

In most of the archaea, the number of the dark matter genes scales super-linearly with the genome size (Figure 1C) but two groups stand out in having inordinately high fractions of unannotated genes, DPANN and Thaumarchaea. The Asgard group contributes two more extreme outliers with much more dark matter than expected from their genome size (Lokiarchaeota archaeon CR_4 and Candidatus Heimdallarchaeota archaeon LC_3).

The protein sequences in the dark matter clusters tend to be much shorter compared with those that are functionally characterized (Figure 1D). The dark matter clusters have the median length of 93 amino acids, compared with 239 and 221 amino acids, respectively, for the ‘gray' and ‘bright' matter clusters. The small size of numerous dark matter genes is likely to result from a combination of at least five factors: (1) some of these ORFs are spurious and do not correspond to actual proteins, (2) the sensitivity of sequence similarity search drops with the protein size, so that short proteins are more likely to end up as dark matter, (3) proteins associated with certain functions, in particular, virus-encoded proteins, tend to be particularly small, (4) small proteins with a narrow phyletic distribution are less likely to attract attention of researchers, and therefore, are, in general, poorly characterized [6], (5) fast evolving lineages such as DPANN tend to have smaller ORFs.

Despite the lack of functional annotation, dark matter genes are far from being a completely random assemblage. This non-randomness can be gleaned from their spatial distribution in archaeal genomes. The 285 155 archaeal dark matter genes form 192 245 ‘islands' (contiguous blocks) in the 524 genomes, with the length of these islands distributed according to a power law-like heavy-tailed distribution (Figure 1E). The longest ‘dark matter island' consists of 49 genes and 205 islands (0.1%) are longer than 10 genes. In contrast, repeated sampling of 285 155 random genes leads to an exponentially declining the distribution of island lengths, with none exceeding 10 genes (frequency of <0.00005%).

## Uncharacterized conserved proteins

An important minority of the dark matter genes encodes uncharacterized conserved proteins. Among the 218 genes in the pan-archaeal core, only one remains uncharacterized (arCOG04076, DUF359 protein family). Analysis of domain fusions suggests that proteins of this family are involved in CoA biosynthesis. However, there are many more functionally uncharacterized genes, with a broad distribution among archaea and often archaea-specific, that have been assigned to the common ancestor of all extant archaea, with a greater than 90% posterior probability [27]. These genes are expected to be involved in essential cellular processes and are prime targets for experimental study (Table 1). Although the structures of many of these proteins have been solved, only a few of them, in addition to DUF359, could be linked to a known pathway or cellular system, based on domain fusions, context analysis or similarity searches.

**Table 1:** Uncharacterized proteins, top priority candidates for experimental study

arCOG or cluster	Representative locus tag	Number of genomes	Comments
All archaea (524 genomes)			
arCOG01159	TK2157	492	Coiled-coil protein; linked to arCOG01158, phosphoserine phosphatase SerB
arCOG01224	TK1195	463	DUF357 family; tightly linked to arCOG02119 (DUF555 family) and Cytidylyltransferase TagD; PDB:2OO2
arCOG04076	TK1697	454	DUF359 family; predicted to be involved in CoA biosynthesis
arCOG04051	TK1296	441	DUF424 family; linked to translational genes; PDB:2QYA
arCOG01336	TK0174	429	AMMECR1 family; linked to arCOG04290, PIN- and Zn ribbon domains; PDB:1VAJ [48]
arCOG04171	TK2293	368	General house-keeping gene context
arCOG04308	TK2131	336	Linked to arCOG00578, Uncharacterized Zn finger containing protein; PDB: 2QZG
arCOG01917	TK0022	392	Zn ribbon domain-containing protein
arCOG02177	TK0743	343	Membrane protein implicated in membrane remodeling or vesicle formation [9]
arCOG04373	TK0173	313	YqgV/DUF77 family; possible thiamine binding protein; PDB:1LXN [49]
arCOG02884	HVO_2173	293	Membrane protein with extracellular Ig-like domain, predicted component of a putative secretion system [9]
arCOG04140	TK1882	252	PDB: 2X3D
arCOG01907	TK0182	245	AIM24 family; PDB: 1PG6
Asgard (8 genomes)			
cls.008013	Lokiarch_14920	7[0]*	Related to Villin-1/gelsolin, predicted actin-binding protein; PDB:3FG7
cls.011087	Lokiarch_54080	7[0]	Membrane protein
DPANN (67 genomes)			
cls.004306	NEQ255	43[0]	Secreted protein, often encoded next to arCOG02487, a predicted component of secretion system and S-layer-like proteins
cls.004259	NEQ050	36[2]	MNT fused to HEPN, usually components of toxin–antitoxin systems, but typically in house-keeping context in DPANN
cls.004340	NEQ484	35[0]	Alpha helical protein, typically in house-keeping context
cls.004634	CMH64_01370	34[0]	Distantly related to YPEB or double PepSY-like domain-containing protein, an inhibitor of protease activity; PDB: 3NQZ [30,31]
Thaumarchaeota (30 genomes)			
arCOG08720	Nmar_1229	29 [0]	Metal-binding protein, DUF2024 family
arCOG08729	Nmar_1451	29 [0]	RHH C-terminal domain, possibly DNA-binding protein
arCOG08818	Nmar_1679	29 [0]	Membrane protein
arCOG08730	Nmar_1445	29 [0]	Zn-binding protein
arCOG08809	Nmar_1788	29 [0]	Membrane protein; likely co-transcribed with DNA replication initiation complex subunit, GINS15 family (arCOG00551)
arCOG08683	Nmar_0539	29 [0]	Membrane protein; likely co-transcribed with galactose-1-phosphate uridylyltransferase (arCOG00422)
arCOG08672	Nmar_1506	29 [0]	Membrane protein
arCOG08761	Nmar_0502	29 [0]	
arCOG08763	Nmar_0508	29 [0]	
arCOG08080	Nmar_1190	29 [0]	
arCOG08668	Nmar_0643	29 [0]	
arCOG08751	Nmar_0717	29 [0]	Likely co-transcribed with membrane associated Zn finger protein (arCOG08750)
arCOG08739	Nmar_0528	29 [0]	
arCOG08727	Nmar_1042	29 [0]	
arCOG08666	Nmar_0410	29 [0]	
arCOG08745	Nmar_0373	29 [0]	

Notes: archaea-specific arCOGs are underlined, clusters (cls) are new groups of orthologs that could not be assigned to previous version of arCOGs.

* Number of archaeal genomes where this gene is present outside of this lineage. Detailed information about these families is available at ftp://ftp.ncbi.nih.gov/pub/wolf/_suppl/archDark2018/.

Asgard, DPANN and Thaumarchaea are especially rich in ‘dark matter' genes, which is not surprising because these are deep-branching, poorly characterized and mostly uncultured archaeal groups (Figure 1C). Despite this dark matter enrichment, there are only a few uncharacterized phylum-specific gene in Asgard and DPANN. In Asgard archaea (eight sequenced genomes), ∼500 arCOGs are represented in seven or eight genomes, and only three among these could not be assigned to arCOGs of the 2014 version [16] (Table 1). One of these three new arCOGs is the Vps25 subunit of the ESCRT-II complex that is implicated in cell division and/or membrane remodeling. The other was initially ‘uncharacterized', but HHpred search shows that one of these is a distant homolog of the eukaryotic signature protein gelsolin, an actin-binding protein. The Asgard archaea encode multiple gelsolin paralogs, but the proteins of this particular cluster were annotated as ‘hypothetical' because of extreme sequence divergence [3]. The paucity of Asgard-specific genes appears surprising because it could be expected that many eukaryotic signature genes found in these genomes would be ancestral and present in most or all Asgard genomes. This is, apparently, not the case although the Asgard genomes are still in the draft stage, so that some genes are likely to be missing.

The paucity of genes specific to the DPANN group (only 47 gene clusters are present in at least 17 out of 68 DPANN genomes and nowhere else) is less puzzling because most of these genomes are streamlined and lack many genes from the archaeal core. For two of the DPANN-specific gene clusters, cls.004259 and cls.004634, HHpred searches reveal similarity to minimal nucleotidyltransferase (MNT) and HEPN domains, and PepSY domain, respectively (Table 1), so these genes can be more appropriately classified as ‘gray', with a general functional prediction. The cls.004259 cluster, most probably, is a toxin–antitoxin module [28,29]. However, the unusual conservation of this gene, compared with the patchy distribution typical of most toxin–antitoxin systems, suggests that this protein plays some important role in the DPANN archaea. The YpeB, or double PepSY-like domain-containing proteins of the cls.004634 is likely to be an inhibitor of proteases that remain to be identified [30,31]. Two other DPANN-specific protein families remain enigmatic (Table 1).

In contrast, there are 53 Thaumarchaea-specific, functionally uncharacterized arCOGs that are represented in at least 90% of the thaumarchaeal genomes and absent from other archaea (Table 1). At present, sequence and genomic context analysis are not highly productive in the elucidation of the likely functions of these genes but they, obviously, are an important resource for experimental study.

## Genomic islands and dark matter genes

Evidence is accumulating that processes of recombination and HGT in prokaryotes are not random [32,33]. Rather, these processes lead to the formation of islands of genes that are linked by common functional themes and/or evolutionary themes. Such islands include clusters or superoperons of house-keeping genes or superoperons [34], defense islands [35,36], islands of integrated elements [37–39], polymorphic toxins [13], virulence islands [40], and others [41]. The formation of genomic islands is driven, in part, by the selective advantages of the spatial clustering of functionally connected genes, such as the possibility of co-regulation, and in part, by non-adaptive ‘preferential attachment’ of non-essential genes, such as defense systems and mobilome components.

Analysis of genomic islands allows us to move many dark matter genes to the ‘gray' zone but often provides even for precise functional predictions. In particular, certain families of house-keeping protein families that evolve fast are retained in stable genomic contexts across long spans of genome evolution. Such a mismatch between sequence and positional conservation has been demonstrated, for example, for DNA replication initiation complex subunits of the GINS and Cdc48 families [42] and for the membrane insertase YidC [9]. Defense islands often include many dark matter genes, and for some of these, involvement in defense functions has been experimentally demonstrated or predicted by sequence analysis [25,36]. Numerous genomic islands that are rich in dark matter actually are integrated mobile genetic elements, such as casposons, proviruses and plasmids in archaea, and in many cases, their precise or approximate boundaries can be identified [14,15,26,43]. Fast evolving multigene systems often contain signature genes that are relatively well conserved, so that the location of such genes can point to the functions of the surrounding dark matter genes. Notable cases in point are CRISPR–Cas systems, with *cas1* genes as a signature [44], viruses with capsid proteins as a signature [45], polymorphic toxins with Zn-dependent proteases of the DUF4157 family as a signature [13], and many others.

**Figure 2.**
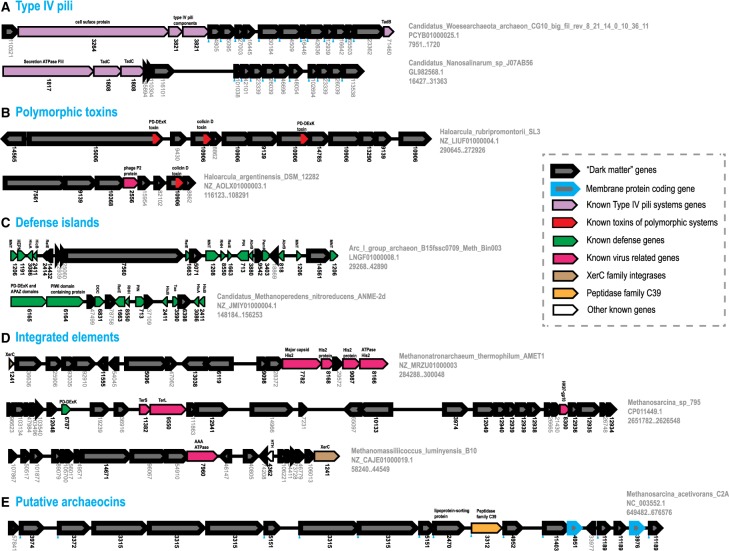
Genomic islands enriched in ‘dark matter’ genes. Genes are shown by block arrows with the length roughly proportional to the size of the corresponding gene. For each gene, the arCOG number (bold) or new protein cluster number (gray) is indicated underneath the respective arrows. These numbers correspond to the assignments available on the ftp site (ftp://ftp.ncbi.nih.gov/pub/wolf/_suppl/archDark2018/). Signal peptides are indicated by blue triangles. For each genomic island, the organism name, genome partition accession number and co-ordinates of the locus are indicated on the right. Brief annotations of the proteins are shown above the arrows. Abbreviation and additional information for some genes: TerS and TerL: terminase small and large subunit, respectively; TadC and TadB: Tad secretion system, secretion accessory proteins C and B; antitoxins: HTH (helix turn helix) protein, RHH (ribbon–helix–helix) proteins; AbrB; MNT, minimal nucleotidyltransferase; toxins: ribonucleases HEPN, PIN, RelE, Txe, PemK, HicA, ribosome interacting toxin Doc; PD-DExK, restriction family endonuclease.

Figure 2 shows five examples of diverse islands in archaeal genomes containing multiple uncharacterized genes for which at least general functional predictions were feasible. Archaeal Type IV pili systems have been explored in detail, uncovering enormous diversity and signs of fast evolution, especially, in the case of pilins [10]. Therefore, it is not surprising that, in some of the DPANN genomes, predicted components of these systems do not show detectable similarity to the respective components from other archaea. Nevertheless, the presence of previously described components of Type IV pili systems, such as FliI, TadC and specific surface proteins, and the presence of signal peptides in the dark matter proteins suggest that all these proteins are diverse pilins (Figure 2A). Colicin D is one of the widespread toxins found in many polymorphic toxin systems in bacteria, often as a C-terminal domain that is fused to other domains involved in the toxin delivery [13]. Thus, the loci shown in Figure 2B, most probably, represent polymorphic toxin systems. The presence of PD-DExK nuclease domains, which also are abundant toxins in these systems, supports this conclusion. Multiple defense islands in archaeal genomes have been thoroughly studied because they contain CRISPR–Cas system components [46]. Many of the remaining ones include multiple TA systems consisting of two small genes that form a two-gene operon [29]. For such operons, if either toxin or antitoxin is known, it is most likely that the other, unannotated small gene in the operon is the respective counterpart (Figure 2C). Other genes present in these loci could be other, yet uncharacterized defense systems. The integrated MGE shown in Figure 2D corresponds to three distinct groups of viruses as indicated by the presence of the respective signature, namely, Pleolipoviridae (His2 major capsid protein), Caudovirales (terminase small and large subunits, TerS and TerL), Fuselloviridae (AAA ATPase, arCOG07960) [45]. All these elements contain numerous uncharacterized genes, supposedly, virion components, genes involved in viral replication, multiple inhibitors of host defense systems, etc. Even if prediction of specific function for most of these genes is currently out of reach, most of them can be confidently annotated as virus-related genes. The final example (Figure 2E) could represent a bacteriocin-like toxin or a quorum-sensing system. One of the genes encoded in this locus encodes a family C39 peptidase involved in bacteriocin precursor peptide processing [47]. Many of the other proteins encoded in this locus contain a leader peptide terminated by a double-glycine motif which is the characteristic recognition substrate of the peptidase [47]. Several genes in this locus are duplicated which is typical of systems involved in interspecies conflicts [13].

## Prospects and outlook

The genomic dark matter of archaeal and bacterial genomes presents both challenges and opportunities for research in microbial biology. Given that the fraction of dark matter remains (nearly) constant as new genomes are sequenced, the total amount and diversity of the dark matter increases rapidly with the growth of the genome database. Thus, there are more and more uncharacterized genes and also a greater capacity to infer their functions using increasingly efficient methods for sequence and genomic context analysis. To make the study of the dark matter informative and productive, carefully curated databases of gene families and improved transfer of annotations are essential. The computational analyses set the stage for systematic experimental investigation. Concerted effort on the functional characterization of the dark matter is likely to bring major pay-offs through improved understanding of poorly studied but crucially important aspects of microbial biology, primarily, various types of intergenomic conflicts and host–parasite coevolution. These processes are especially poorly understood in archaea, making the study of the dark matter particularly pertinent. Moreover, there is potential for the discovery of new defense systems that could be subsequently adopted as genome engineering tools as amply demonstrated by the discovery of different variants of CRISPR–Cas systems.

## Abbreviations

arCOGs: archaeal clusters of orthologous genes
DPANN: Diapherotrites, Parvarchaeota, Aenigmarchaeota, Nanoarchaeota and Nanohaloarchaeota
HTH: helix turn helix
MNT: minimal nucleotidyltransferase
ORFs: open reading frames
RHH: ribbon–helix–helix
TACK: Thaumarchaea, Aigarchaea, Crenarchaea, Korarchaea

## References

[1] Castelle C.J. and Banfield J.F. (2018) Major new microbial groups expand diversity and alter our understanding of the tree of life. Cell 172, 1181–1197 10.1016/j.cell.2018.02.016 29522741

[2] Adam P.S., Borrel G., Brochier-Armanet C. and Gribaldo S. (2017) The growing tree of Archaea: new perspectives on their diversity, evolution and ecology. ISME J. 11, 2407–2425 10.1038/ismej.2017.122 28777382 PMC5649171

[3] Zaremba-Niedzwiedzka K., Caceres E.F., Saw J.H., Bäckström D., Juzokaite L., Vancaester E. et al. (2017) Asgard archaea illuminate the origin of eukaryotic cellular complexity. Nature 541, 353–358 10.1038/nature21031 28077874

[4] Sorokin D.Y., Makarova K.S., Abbas B., Ferrer M., Golyshin P.N., Galinski E.A. et al. (2017) Discovery of extremely halophilic, methyl-reducing euryarchaea provides insights into the evolutionary origin of methanogenesis. Nat. Microbiol. 2, 17081 10.1038/nmicrobiol.2017.81 28555626 PMC5494993

[5] Bork P. (2000) Powers and pitfalls in sequence analysis: the 70% hurdle. Genome Res. 10, 398–400 10.1101/gr.10.4.398 10779480

[6] Storz G., Wolf Y.I. and Ramamurthi K.S. (2014) Small proteins can no longer be ignored. Annu. Rev. Biochem. 83, 753–777 10.1146/annurev-biochem-070611-102400 24606146 PMC4166647

[7] Márquez V., Fröhlich T., Armache J.P., Sohmen D., Dönhöfer A., Mikolajka A. et al. (2011) Proteomic characterization of archaeal ribosomes reveals the presence of novel archaeal-specific ribosomal proteins. J. Mol. Biol. 405, 1215–1232 10.1016/j.jmb.2010.11.055 21134383

[8] Wu P., Brockenbrough J.S., Paddy M.R. and Aris J.P. (1998) NCL1, a novel gene for a non-essential nuclear protein in *Saccharomyces cerevisiae*. Gene 220, 109–117 10.1016/S0378-1119(98)00330-8 9767141

[9] Makarova K.S., Galperin M.Y. and Koonin E.V. (2015) Comparative genomic analysis of evolutionarily conserved but functionally uncharacterized membrane proteins in archaea: prediction of novel components of secretion, membrane remodeling and glycosylation systems. Biochimie 118, 302–312 10.1016/j.biochi.2015.01.004 25583072 PMC5898192

[10] Makarova K.S., Koonin E.V. and Albers S.V. (2016) Diversity and evolution of type IV pili systems in Archaea. Front. Microbiol. 7, 667 10.3389/fmicb.2016.00667 27199977 PMC4858521

[11] Makarova K.S., Galperin M.Y. and Koonin E.V. (2017) Proposed role for KaiC-like ATPases as major signal transduction hubs in Archaea. mBio 8, e01959-17 10.1128/mBio.01959-17 29208747 PMC5717392

[12] Galperin M.Y., Makarova K.S., Wolf Y.I. and Koonin E.V. (2018) Phyletic distribution and lineage-specific domain architectures of archaeal two-component signal transduction systems. J. Bacteriol. 200, e00681-17 10.1128/JB.00681-17 29263101 PMC5847659

[13] Zhang D., de Souza R.F., Anantharaman V., Iyer L.M. and Aravind L. (2012) Polymorphic toxin systems: comprehensive characterization of trafficking modes, processing, mechanisms of action, immunity and ecology using comparative genomics. Biol. Direct 7, 18 10.1186/1745-6150-7-18 22731697 PMC3482391

[14] Forterre P., Krupovic M., Raymann K. and Soler N. (2014) Plasmids from Euryarchaeota. Microbiol. Spectr. 2, PLAS-0027-2014. 10.1128/microbiolspec.PLAS-0027-2014 26104461

[15] Yutin N., Bäckström D., Ettema T.J.G., Krupovic M. and Koonin E.V. (2018) Vast diversity of prokaryotic virus genomes encoding double jelly-roll major capsid proteins uncovered by genomic and metagenomic sequence analysis. Virol. J. 15, 67 10.1186/s12985-018-0974-y 29636073 PMC5894146

[16] Makarova K.S., Wolf Y.I. and Koonin E.V. (2015) Archaeal clusters of orthologous genes (arCOGs): an update and application for analysis of shared features between thermococcales, methanococcales, and methanobacteriales. Life 5, 818–840 10.3390/life5010818 25764277 PMC4390880

[17] Hanson A.D., Pribat A., Waller J.C. and de Crécy-Lagard V. (2010) ‘Unknown’ proteins and ‘orphan’ enzymes: the missing half of the engineering parts list–and how to find it. Biochem. J. 425, 1–11 10.1042/BJ20091328 PMC3022307 20001958

[18] Ellens K.W., Christian N., Singh C., Satagopam V.P., May P. and Linster C.L. (2017) Confronting the catalytic dark matter encoded by sequenced genomes. Nucleic Acids Res. 45, 11495–11514 10.1093/nar/gkx937 29059321 PMC5714238

[19] Galperin M.Y. and Koonin E.V. (2010) From complete genome sequence to ‘complete’ understanding? Trends Biotechnol. 28, 398–406 10.1016/j.tibtech.2010.05.006 20647113 PMC3065831

[20] Galperin M.Y. and Koonin E.V. (2000) Who's your neighbor? New computational approaches for functional genomics. Nat. Biotechnol. 18, 609–613 10.1038/76443 10835597

[21] Niehaus T.D., Thamm A.M., de Crécy-Lagard V. and Hanson A.D. (2015) Proteins of unknown biochemical function: a persistent problem and a roadmap to help overcome it. Plant Physiol. 169, 1436–1442 10.1104/pp.15.00959 26269542 PMC4634069

[22] Chang Y.C., Hu Z., Rachlin J., Anton B.P., Kasif S., Roberts R.J. et al. (2016) COMBREX-DB: an experiment centered database of protein function: knowledge, predictions and knowledge gaps. Nucleic Acids Res. 44, D330–D335 10.1093/nar/gkv1324 26635392 PMC4702925

[23] Vetting M.W., Al-Obaidi N., Zhao S., San Francisco B., Kim J., Wichelecki D.J. et al. (2015) Experimental strategies for functional annotation and metabolism discovery: targeted screening of solute binding proteins and unbiased panning of metabolomes. Biochemistry 54, 909–931 10.1021/bi501388y 25540822 PMC4310620

[24] Gerlt J.A., Allen K.N., Almo S.C., Armstrong R.N., Babbitt P.C., Cronan J.E. et al. (2011) The enzyme function initiative. Biochemistry 50, 9950–9962 10.1021/bi201312u 21999478 PMC3238057

[25] Doron S., Melamed S., Ofir G., Leavitt A., Lopatina A., Keren M. et al. (2018) Systematic discovery of antiphage defense systems in the microbial pangenome. Science 359, eaar4120 10.1126/science.aar4120 29371424 PMC6387622

[26] Makarova K.S., Wolf Y.I., Forterre P., Prangishvili D., Krupovic M. and Koonin E.V. (2014) Dark matter in archaeal genomes: a rich source of novel mobile elements, defense systems and secretory complexes. Extremophiles 18, 877–893 10.1007/s00792-014-0672-7 25113822 PMC4158269

[27] Wolf Y.I., Makarova K.S., Yutin N. and Koonin E.V. (2012) Updated clusters of orthologous genes for *Archaea*: a complex ancestor of the Archaea and the byways of horizontal gene transfer. Biol. Direct 7, 46 10.1186/1745-6150-7-46 23241446 PMC3534625

[28] Jia X., Yao J., Gao Z., Liu G., Dong Y.H., Wang X. et al. (2018) Structure-function analyses reveal the molecular architecture and neutralization mechanism of a bacterial HEPN-MNT toxin-antitoxin system. J. Biol. Chem. 293, 6812–6823 10.1074/jbc.RA118.002421 29555683 PMC5936836

[29] Makarova K.S., Wolf Y.I. and Koonin E.V. (2009) Comprehensive comparative-genomic analysis of type 2 toxin-antitoxin systems and related mobile stress response systems in prokaryotes. Biol. Direct 4, 19 10.1186/1745-6150-4-19 19493340 PMC2701414

[30] Gao X., Wang J., Yu D.Q., Bian F., Xie B.B., Chen X.L. et al. (2010) Structural basis for the autoprocessing of zinc metalloproteases in the thermolysin family. Proc. Natl Acad. Sci. U.S.A. 107, 17569–17574 10.1073/pnas.1005681107 20876133 PMC2955107

[31] Yeats C., Rawlings N.D. and Bateman A. (2004) The PepSY domain: a regulator of peptidase activity in the microbial environment? Trends Biochem. Sci. 29, 169–172 10.1016/j.tibs.2004.02.004 15124630

[32] Touchon M. and Rocha E.P. (2016) Coevolution of the organization and structure of prokaryotic genomes. Cold Spring Harb. Perspect. Biol. 8, a018168 10.1101/cshperspect.a018168 26729648 PMC4691797

[33] Koonin E.V. (2009) Evolution of genome architecture. Int. J. Biochem. Cell Biol. 41, 298–306 10.1016/j.biocel.2008.09.015 18929678 PMC3272702

[34] Rogozin I.B., Makarova K.S., Murvai J., Czabarka E., Wolf Y.I., Tatusov R.L. et al. (2002) Connected gene neighborhoods in prokaryotic genomes. Nucleic Acids Res. 30, 2212–2223 10.1093/nar/30.10.2212 12000841 PMC115289

[35] Makarova K.S., Wolf Y.I., Snir S. and Koonin E.V. (2011) Defense islands in bacterial and archaeal genomes and prediction of novel defense systems. J. Bacteriol. 193, 6039–6056 10.1128/JB.05535-11 21908672 PMC3194920

[36] Makarova K.S., Wolf Y.I. and Koonin E.V. (2013) Comparative genomics of defense systems in archaea and bacteria. Nucleic Acids Res. 41, 4360–4377 10.1093/nar/gkt157 23470997 PMC3632139

[37] Hurwitz B.L., Ponsero A., Thornton J. Jr and U'Ren J.M. (2018) Phage hunters: Computational strategies for finding phages in large-scale ‘omics datasets. Virus Res. 244, 110–115 10.1016/j.virusres.2017.10.019 29100906

[38] Johnson C.M. and Grossman A.D. (2015) Integrative and conjugative elements (ICEs): what they do and how they work. Annu. Rev. Genet. 49, 577–601 10.1146/annurev-genet-112414-055018 26473380 PMC5180612

[39] Grazziotin A.L., Koonin E.V. and Kristensen D.M. (2017) Prokaryotic virus orthologous groups (pVOGs): a resource for comparative genomics and protein family annotation. Nucleic Acids Res. 45, D491–D498 10.1093/nar/gkw975 27789703 PMC5210652

[40] Pallen M.J. and Wren B.W. (2007) Bacterial pathogenomics. Nature 449, 835–842 10.1038/nature06248 17943120

[41] Langille M.G., Hsiao W.W. and Brinkman F.S. (2010) Detecting genomic islands using bioinformatics approaches. Nat. Rev. Microbiol. 8, 373–382 10.1038/nrmicro2350 20395967

[42] Makarova K.S. and Koonin E.V. (2013) Archaeology of eukaryotic DNA replication. Cold Spring Harb. Perspect. Biol. 5, a012963 10.1101/cshperspect.a012963 23881942 PMC3809583

[43] Krupovic M., Makarova K.S., Forterre P., Prangishvili D. and Koonin E.V. (2014) Casposons: a new superfamily of self-synthesizing DNA transposons at the origin of prokaryotic CRISPR-Cas immunity. BMC Biol. 12, 36 10.1186/1741-7007-12-36 24884953 PMC4046053

[44] Makarova K.S., Wolf Y.I., Alkhnbashi O.S., Costa F., Shah S.A., Saunders S.J. et al. (2015) An updated evolutionary classification of CRISPR-Cas systems. Nat. Rev. Microbiol. 13, 722–736 10.1038/nrmicro3569 26411297 PMC5426118

[45] Krupovic M., Cvirkaite-Krupovic V., Iranzo J., Prangishvili D. and Koonin E.V. (2018) Viruses of archaea: structural, functional, environmental and evolutionary genomics. Virus Res. 244, 181–193 10.1016/j.virusres.2017.11.025 29175107 PMC5801132

[46] Shmakov S.A., Makarova K.S., Wolf Y.I., Severinov K.V. and Koonin E.V. (2018) Systematic prediction of genes functionally linked to CRISPR-Cas systems by gene neighborhood analysis. Proc. Natl Acad. Sci. U.S.A. 115, E5307–E5316 10.1073/pnas.1803440115 29784811 PMC6003329

[47] Havarstein L.S., Diep D.B. and Nes I.F. (1995) A family of bacteriocin ABC transporters carry out proteolytic processing of their substrates concomitant with export. Mol. Microbiol. 16, 229–240 10.1111/j.1365-2958.1995.tb02295.x 7565085

[48] Vitelli F., Piccini M., Caroli F., Franco B., Malandrini A., Pober B. et al. (1999) Identification and characterization of a highly conserved protein absent in the Alport syndrome (A), mental retardation (M), midface hypoplasia (M), and elliptocytosis (E) contiguous gene deletion syndrome (AMME). Genomics 55, 335–340 10.1006/geno.1998.5666 10049589

[49] Dermoun Z., Foulon A., Miller M.D., Harrington D.J., Deacon A.M., Sebban-Kreuzer C. et al. (2010) TM0486 from the hyperthermophilic anaerobe *Thermotoga maritima* is a thiamin-binding protein involved in response of the cell to oxidative conditions. J. Mol. Biol. 400, 463–476 10.1016/j.jmb.2010.05.014 20471400 PMC2980899

