# Supplementary material for: Towards functional characterization of archaeal genomic dark matter
Source: Biochem Soc Trans. 2019 Feb 1;47(1):389–98. doi: 10.1042/BST20180560 (PMC6393860; doi:10.1042/BST20180560)
Supplement: Supplementary Tables [file BST-47-389-s1.pdf]

Supplementary table 1. Genomes analyzed in this work and the dark matter fraction.

Note: Proteins which were annotated as "hypothertical", "unknown" and "uncharacterized" were counted for collumn E data. Proteins assined to arCOGs with category "S" were counted for column F data. The genomes colored in blue were omitted in order to assess the average fraction of dark matter gene content in archaeal genomes reported in the text.

FTP accession number	Genome Name	Archaeal lineage	Number of proteins	Fraction of dark matter in the submitted annotation
GCA_001563335.1	Candidatus_Thorarchaeota_archaeon_SMTZ1-45_	Asgard	3208	91%
GCA_001761425.1	Nanohaloarchaea_archaeon_SG9_	DPANN	1183	90%
GCA_001563325.1	Candidatus_Thorarchaeota_archaeon_SMTZ1-83_	Asgard	3029	89%
GCA_001872325.1	Candidatus_Pacearchaeota_archaeon_CG1_02_35_32_	DPANN	1013	88%
GCA_001595815.1	Theionarchaea_archaeon_DG-70-1_	Theionarchaea	4270	87%
GCA_001595795.1	Theionarchaea_archaeon_DG-70_	Theionarchaea	3483	86%
GCA_001871495.1	Candidatus_Micrarchaeota_archaeon_CG1_02_51_15_	DPANN	1244	86%
GCA_001872315.1	Candidatus_Pacearchaeota_archaeon_CG1_02_39_14_	DPANN	807	85%
GCA_001872795.1	Candidatus_Pacearchaeota_archaeon_CG1_02_32_132_	DPANN	872	84%
GCA_001918715.1	Crenarchaeota_archaeon_13_1_40CM_3_53_5_	unclassified	2419	84%
GCA_001872125.1	Candidatus_Pacearchaeota_archaeon_CG1_02_30_18_	DPANN	660	83%
GCA_001871595.1	Candidatus_Micrarchaeota_archaeon_CG1_02_55_22_	DPANN	1162	83%
GCA_001786415.1	Candidatus_Pacearchaeota_archaeon_RBG_19FT_COMBO_34_9	DPANN	645	83%
GCA_001787285.1	Candidatus_Pacearchaeota_archaeon_RBG_13_33_26_	DPANN	677	82%
GCA_001273385.1	miscellaneous_Crenarchaeota_group-6_archaeon_AD8-1_	Bathyarchaeota	1505	82%
GCA_001873985.1	Candidatus_Aenigmarchaeota_archaeon_CG1_02_38_14_	DPANN	1011	82%
GCA_002172575.1	Euryarchaeota_archaeon_TMED103_	ucEuryarchaeota	1636	81%
GCA_001871655.1	Candidatus_Micrarchaeota_archaeon_CG1_02_60_51_	DPANN	849	81%
GCA_001889985.1	Candidatus_Micrarchaeum_acidiphilum_ARMAN-1_	DPANN	1035	81%
GCA_001871605.1	Candidatus_Micrarchaeota_archaeon_CG1_02_55_41_	DPANN	768	80%
GCA_002204695.1	Thermoplasmatales_archaeon_ARMAN_	Thermoplasmata	991	79%
GCA_001856845.1	Thermoplasmatales_archaeon_Gpl_	Thermoplasmata	1923	79%
GCA_000496135.1	Thermoplasmatales_archaeon_E-plasma_	Thermoplasmata	1674	78%
GCA_001872145.1	Candidatus_Pacearchaeota_archaeon_CG1_02_31_27_	DPANN	779	78%
GCA_002763165.1	Candidatus_Pacearchaeota_archaeon_CG10_big_fil_rev_8_21_1	DPANN	1115	77%
GCA_000220375.1	Candidatus_Nanosalina_sp_J07AB43_	DPANN	1677	58%
GCA_000730285.1	Candidatus_Nitrososphaera_evergladensis_SR1_	Thaumarchaeota	3499	52%
GCA_000303155.1	Candidatus_Nitrososphaera_gargensis_Ga9_2_	Thaumarchaeota	3565	53%
GCA_001940655.1	Candidatus_Lokiarchaeota_archaeon_CR_4_	Asgard	4413	62%
GCA_001940645.1	Candidatus_Heimdallarchaeota_archaeon_LC_3_	Asgard	5514	68%
GCA_000200715.1	Cenarchaeum_symbiosum_A_	Thaumarchaeota	2017	51%
GCA_002794195.1	Candidatus_Pacearchaeota_archaeon_CG10_big_fil_rev_8_21_1	DPANN	907	71%
GCA_000722915.1	Marine_Group_I_thaumarchaeote_SCGC_AAA799-N04_	Thaumarchaeota	1767	41%
GCA_000220355.1	Candidatus_Nanosalinarum_sp_J07AB56_	DPANN	1407	53%
GCA_002762735.1	Candidatus_Pacearchaeota_archaeon_CG11_big_fil_rev_8_21_1	DPANN	784	74%
GCF_000698785.1	Nitrososphaera_viennensis_EN76	Thaumarchaeota	2801	57%
GCA_000875775.1	Candidatus_Nitrosopumilus_piranensis_D3C	Thaumarchaeota	2161	51%

GCA_002687825.1	Nanoarchaeota_archaeon_	DPANN	935	72%
GCA_002778455.1	Candidatus_Micrarchaeota_archaeon_CG10_big_fil_rev_8_21_1	DPANN	1144	67%
GCA_000416085.1	halophilic_archaeon_J07HX64_	Halobacteria	3026	49%
GCA_002789435.1	Candidatus_Pacearchaeota_archaeon_CG_4_9_14_0_8_um_filte	DPANN	951	74%
GCA_002763075.1	Candidatus_Pacearchaeota_archaeon_CG10_big_fil_rev_8_21_1	DPANN	1077	75%
GCA_000746745.1	Marine_Group_I_thaumarchaeote_SCGC_RSA3_	Thaumarchaeota	2412	39%
GCA_002792635.1	Candidatus_Pacearchaeota_archaeon_CG_4_10_14_0_2_um_filt	DPANN	950	74%
GCA_002792675.1	Candidatus_Pacearchaeota_archaeon_CG_4_10_14_0_2_um_filt	DPANN	618	71%
GCA_002788615.1	Candidatus_Pacearchaeota_archaeon_CG_4_9_14_0_2_um_filte	DPANN	931	74%
GCA_000724145.1	Marine_Group_I_thaumarchaeote_SCGC_AAA799-E16_	Thaumarchaeota	1910	36%
GCA_002794155.1	Candidatus_Pacearchaeota_archaeon_CG10_big_fil_rev_8_21_1	DPANN	902	75%
GCA_002790875.1	Candidatus_Pacearchaeota_archaeon_CG_4_9_14_3_um_filter_	DPANN	740	73%
GCA_002790905.1	Candidatus_Pacearchaeota_archaeon_CG_4_9_14_3_um_filter_	DPANN	986	75%
GCA_002784645.1	Candidatus_Pacearchaeota_archaeon_CG_4_10_14_0_8_um_filt	DPANN	953	74%
GCA_000299365.1	Candidatus_Nitrosopumilus_koreensis_AR1	Thaumarchaeota	1890	47%
GCA_002763155.1	Candidatus_Pacearchaeota_archaeon_CG10_big_fil_rev_8_21_1	DPANN	780	71%
GCA_000746765.1	Marine_Group_I_thaumarchaeote_SCGC_AAA799-D11_	Thaumarchaeota	1732	37%
GCA_900065925.1	Candidatus_Nitrosotalea_devanaterra_	Thaumarchaeota	2103	44%
GCA_002762795.1	Candidatus_Woesearchaeota_archaeon_CG10_big_fil_rev_8_21	DPANN	1463	75%
GCA_900036045.1	Methanoculleus_sp_MAB1_	Methanomicrobia	3450	58%
GCA_002794175.1	Candidatus_Pacearchaeota_archaeon_CG10_big_fil_rev_8_21_1	DPANN	729	69%
GCA_002781865.1	Candidatus_Aenigmarchaeota_archaeon_CG15_BIG_FIL_POST_F	DPANN	1049	70%
GCF_000328925.1	Nitrosopumilus_sp_AR	Thaumarchaeota	3898	42%
GCA_002784265.1	Candidatus_Aenigmarchaeota_archaeon_CG_4_10_14_3_um_fil	DPANN	1118	70%
GCA_002792115.1	Candidatus_Woesearchaeota_archaeon_CG_4_10_14_0_2_um_	DPANN	1410	74%
GCA_002791855.1	Candidatus_Aenigmarchaeota_archaeon_CG_4_10_14_0_8_um_	DPANN	1067	70%
GCF_000956175.1	Candidatus_Nitrosopumilus_adriaticus_NF5	Thaumarchaeota	2037	43%
GCF_000328945.1	Nitrosopumilus_sp_SJ	Thaumarchaeota	1853	39%
GCA_002792655.1	Candidatus_Pacearchaeota_archaeon_CG_4_10_14_0_2_um_filt	DPANN	688	66%
GCA_000986845.1	Lokiarchaeum_sp_GC14_75_	Asgard	5384	47%
GCA_000830315.1	archaeon_GW2011_AR20_	DPANN	1010	58%
GCA_000496235.1	uncultured_archaeon_A07HR60_	Halobacteria	2856	40%
GCA_000830295.1	archaeon_GW2011_AR15_	DPANN	1308	57%
GCF_000710605.1	Haloterrigena_jeotgali_A29	Halobacteria	4045	40%
GCA_002782805.1	Candidatus_Aenigmarchaeota_archaeon_CG_4_8_14_3_um_filte	DPANN	1004	69%
GCF_000299395.1	Candidatus_Nitrosopumilus_sediminis_AR2	Thaumarchaeota	1949	41%
GCA_002789635.1	Candidatus_Aenigmarchaeota_archaeon_CG_4_9_14_3_um_filte	DPANN	1092	70%
GCA_002763115.1	Candidatus_Pacearchaeota_archaeon_CG10_big_fil_rev_8_21_1	DPANN	700	67%
GCA_000204585.1	Candidatus_Nitrosoarchaeum_limnia_SFB1	Thaumarchaeota	2038	46%
GCA_002762845.1	Candidatus_Woesearchaeota_archaeon_CG10_big_fil_rev_8_21	DPANN	890	70%
GCF_000337175.1	Natrinema_gari_JCM_14663	Halobacteria	3773	40%
GCA_002762785.1	Candidatus_Woesearchaeota_archaeon_CG10_big_fil_rev_8_21	DPANN	1259	69%
GCA_002763205.1	Candidatus_Micrarchaeota_archaeon_CG10_big_fil_rev_8_21_1	DPANN	798	69%
GCA_000234805.1	Pyrobaculum_ferrireducens_1860	Thermoproteales	2827	48%
GCA_000007225.1	Pyrobaculum_aerophilum_str_IM2	Thermoproteales	2605	47%
GCA_000247545.1	Pyrobaculum_oguniense_TE7	Thermoproteales	2835	41%
GCA_002762985.1	Candidatus_Woesearchaeota_archaeon_CG10_big_fil_rev_8_21	DPANN	1513	70%

GCA_002763255.1	Candidatus_Micrarchaeota_archaeon_CG09_land_8_20_14_0_1	DPANN	865	68%
GCA_002763265.1	archaeon_CG10_big_fil_rev_8_21_14_0_10_43_11_	unclassified	1077	70%
GCF_001541925.1	Nitrosopumilus_sp_Nsub	Thaumarchaeota	1604	38%
GCA_002792915.1	Candidatus_Micrarchaeota_archaeon_CG_4_10_14_0_2_um_filt	DPANN	851	66%
GCA_002780285.1	Candidatus_Pacearchaeota_archaeon_CG06_land_8_20_14_3_0	DPANN	673	68%
GCA_000830275.1	archaeon_GW2011_AR10_	unclassified	1339	54%
GCA_002785565.1	Candidatus_Micrarchaeota_archaeon_CG10_big_fil_rev_8_21_1	DPANN	815	66%
GCF_001625445.1	Haladaptatus_sp_R4	Halobacteria	4130	39%
GCF_000710615.1	Haladaptatus_cibarius_D43	Halobacteria	3843	39%
GCF_001485575.1	Haloarcula_sp_CBA1127	Halobacteria	4130	39%
GCF_002572525.1	Natrinema_sp_CBA1119	Halobacteria	4647	36%
GCA_001443365.1	Thaumarchaeota_archaeon_CSP1-1_	Thaumarchaeota	1652	41%
GCF_000281695.1	Natrinema_sp_J7-2	Halobacteria	3595	38%
GCF_000025625.1	Natrialba_magadii_ATCC_43099	Halobacteria	4076	38%
GCF_002156965.1	Candidatus_Nitrosomarinus_catalina_SPOT01	Thaumarchaeota	1591	36%
GCF_000230715.2	Natronobacterium_gregoryi_SP2	Halobacteria	3588	37%
GCF_000731985.1	Natrinema_altunense_AJ2	Halobacteria	3588	39%
GCA_000246735.1	uncultured_marine_group_II_euryarchaeote_	Thermoplasmata	1781	34%
GCA_001307315.1	Halolamina_pelagica_CDK2	Halobacteria	3485	40%
GCF_001469955.1	Haloprofundus_marisrubri_SB9	Halobacteria	3845	40%
GCF_000337115.1	Haloterrigena_thermotolerans_DSM_11522	Halobacteria	3688	38%
GCF_000337535.1	Natrialba_aegyptia_DSM_13077	Halobacteria	4203	37%
GCF_000230735.2	Natrinema_pellirubrum_DSM_15624	Halobacteria	4096	37%
GCF_001647155.1	Haloarcula_sp_K1	Halobacteria	4131	38%
GCF_000220175.1	Candidatus_Nitrosoarchaeum_koreensis_MY1	Thaumarchaeota	1828	37%
GCF_000025325.1	Haloterrigena_turkmenica_DSM_5511	Halobacteria	4989	35%
GCF_000493245.1	Natrinema_sp_J7-1	Halobacteria	3465	37%
GCF_002494345.1	Natrinema_ejinorense_JCM_13890	Halobacteria	4203	35%
GCF_002906575.1	Salinigranum_rubrum_GX10	Halobacteria	4476	41%
GCF_000217715.1	Halopiger_xanaduensis_SH-6	Halobacteria	4087	36%
GCF_000337195.1	Natrinema_versiforme_JCM_10478	Halobacteria	3962	37%
GCF_000337455.1	Halosimplex_carlsbadense_2-9-1	Halobacteria	4274	40%
GCA_001399795.1	Candidatus_Bathyarchaeota_archaeon_BA2_	Bathyarchaeota	1761	43%
GCF_001971705.1	Haloterrigena_daqingensis_	Halobacteria	3643	36%
GCF_000336615.1	Haloarcula_amylolytica_JCM_13557	Halobacteria	3988	37%
GCF_000336895.1	Haloarcula_argentinensis_DSM_12282	Halobacteria	3981	39%
GCF_000427685.1	Haloplanus_natans_DSM_17983	Halobacteria	3664	41%
GCF_000690595.1	Haloterrigena_mahii_H13	Halobacteria	3591	35%
GCF_000007345.1	Methanosarcina_acetivorans_C2A	Methanomicrobia	4567	39%
GCA_002779235.1	Candidatus_Woesearchaeota_archaeon_CG07_land_8_20_14_0	DPANN	975	65%
GCF_001482285.1	Haloferax_sp_Q22	Halobacteria	3849	36%
GCF_000283335.1	Halogranum_salarium_B-1	Halobacteria	4304	39%
GCF_000011085.1	Haloarcula_marismortui_ATCC_43049	Halobacteria	4144	37%
GCF_000337555.1	Natrialba_asiatica_DSM_12278	Halobacteria	3996	36%
GCF_001989615.1	Halorientalis_sp_IM1011	Halobacteria	3720	40%
GCF_000827835.1	Haloarcula_sp_CBA1115	Halobacteria	3966	36%
GCA_000496215.1	uncultured_archaeon_A07HN63_	Halobacteria	2507	38%

GCA_000416025.1	Halonotius_sp_J07HN6_	Halobacteria	2913	40%
GCF_000337595.1	Natrialba_taiwanensis_DSM_12281	Halobacteria	4135	37%
GCA_001940755.1	Candidatus_Heimdallarchaeota_archaeon_AB_125_	Asgard	2348	59%
GCF_000337275.1	Haloarcula_sinaiiensis_ATCC_33800	Halobacteria	4185	38%
GCF_000337495.1	Haloterrigena_salina_JCM_13891	Halobacteria	4332	36%
GCF_000337475.1	Haloterrigena_limicola_JCM_13563	Halobacteria	3327	37%
GCA_000328525.1	Halovivax_ruber_XH-70	Halobacteria	3099	39%
GCF_000237865.1	Haloquadratum_walsbyi_C23_	Halobacteria	2771	37%
GCA_002763225.1	Candidatus_Diapherotrites_archaeon_CG10_big_fil_rev_8_21_1	DPANN	1100	65%
GCF_002025255.1	Halolamina_sp_CBA1230	Halobacteria	3389	38%
GCA_002763275.1	Candidatus_Micrarchaeota_archaeon_CG09_land_8_20_14_0_1	DPANN	749	64%
GCF_000455365.1	Halopiger_djelfimassiliensis_IIH2	Halobacteria	3560	35%
GCF_000383975.1	Natronorubrum_tibetense_GA33	Halobacteria	4557	39%
GCF_000337515.1	Halovivax_asiaticus_JCM_14624	Halobacteria	3050	38%
GCF_000337715.1	Natronorubrum_bangense_JCM_10635	Halobacteria	3797	37%
GCA_000416065.1	Halonotius_sp_J07HN4_	Halobacteria	3226	39%
GCF_002177135.1	Natronolimnobius_baerhuensis_CGMCC_1_3597	Halobacteria	3654	35%
GCF_000336635.1	Haloarcula_japonica_DSM_6131	Halobacteria	4057	38%
GCA_002779075.1	Candidatus_Diapherotrites_archaeon_CG08_land_8_20_14_0_2	DPANN	782	71%
GCA_002412065.1	Candidatus_Parvarchaeum_acidiphilum_ARMAN-4_	DPANN	911	45%
GCF_000784335.1	Halopiger_salifodinae_KCY07-B2	Halobacteria	3978	34%
GCF_002287175.1	Methanobacterium_bryantii_M_o_H_	Methanobacteria	3204	39%
GCF_000337775.1	Haloarcula_vallismortis_ATCC_29715	Halobacteria	3825	38%
GCF_000970145.1	Methanosarcina_siciliae_C2J	Methanomicrobia	4269	41%
GCF_000306765.2	Haloferax_mediterranei_ATCC_33500_CGMCC_1_2087	Halobacteria	3761	36%
GCF_001861355.1	Natrialba_sp_SSL1	Halobacteria	4149	34%
GCF_000746075.1	Methanobacterium_arcticum_M2	Methanobacteria	3174	41%
GCF_000739575.1	Halobellus_rufus_CBA1103	Halobacteria	3533	39%
GCF_000337415.1	Halorubrum_tebenquichense_DSM_14210	Halobacteria	3113	36%
GCF_000376445.1	Haladaptatus_paucihalophilus_DX253	Halobacteria	4212	36%
GCF_000336915.1	Halococcus_saccharolyticus_DSM_5350	Halobacteria	3325	35%
GCF_000172995.2	Halogeometricum_borinquense_DSM_11551_PR_3	Halobacteria	3774	35%
GCF_000336875.1	Halorubrum_californiensis_DSM_19288	Halobacteria	3313	37%
GCA_002412085.1	Candidatus_Parvarchaeum_acidophilus_ARMAN-5_	DPANN	1042	46%
GCF_000337735.1	Natronorubrum_sulfidifaciens_JCM_14089	Halobacteria	3298	36%
GCF_001280455.1	Halorubrum_tropicale_5	Halobacteria	3373	35%
GCF_000226975.2	Halobiforma_lacisalsi_AJ5	Halobacteria	4088	36%
GCF_001729285.1	Methanobacterium_sp_A39	Methanobacteria	3082	39%
GCF_001485555.1	Haloarcula_sp_CBA1128	Halobacteria	3947	35%
GCF_000336755.1	Haloferax_elongans_ATCC_BAA-1513	Halobacteria	3776	37%
GCF_000969985.1	Methanosarcina_barkeri_str_Wiesmoor	Methanomicrobia	3781	38%
GCF_000196895.1	Halalkalicoccus_jeotgali_B3	Halobacteria	3679	36%
GCF_000745485.1	Methanobacterium_veterum_MK4	Methanobacteria	3156	41%
GCA_001189275.1	Pyrobaculum_sp_WP30	Thermoproteales	2216	38%
GCF_001953745.1	Haloterrigena_saccharevitans_AB14	Halobacteria	3745	33%
GCF_000969945.1	Methanosarcina_sp_Kolksee	Methanomicrobia	3553	36%
GCF_000022205.1	Halorubrum_lacusprofundi_ATCC_49239	Halobacteria	3456	35%

GCF_000379085.1	Halomicrobium_katesii_DSM_19301	Halobacteria	3382	40%
GCF_000337835.1	Haloferax_sulfurifontis_ATCC_BAA-897	Halobacteria	3664	36%
GCF_000517625.1	Halostagnicola_larsenii_XH-48	Halobacteria	3858	35%
GCF_000023965.1	Halomicrobium_mukohataei_DSM_12286	Halobacteria	3232	35%
GCF_000337815.1	Haloferax_mucosum_ATCC_BAA-1512	Halobacteria	3287	36%
GCF_000337915.1	Halorubrum_saccharovorum_DSM_1137	Halobacteria	3122	37%
GCF_000455345.1	Halopiger_goleimassiliensis_IIH3	Halobacteria	3705	36%
GCF_002487355.1	Candidatus_Methanoperedens_sp_BLZ2_	Methanomicrobia	3790	45%
GCF_000337435.1	Halorubrum_terrestre_JCM_10247	Halobacteria	3180	36%
GCF_000970285.1	Methanosarcina_horonobensis_HB-1_JCM_15518	Methanomicrobia	4144	38%
GCA_000007185.1	Methanopyrus_kandleri_AV19	Methanopyri	1687	34%
GCF_002906215.1	Candidatus_Nitrosocaldus_islandicus_	Thaumarchaeota	1641	49%
GCF_001542905.1	Halorubrum_aethiopicum_SAH-A6	Halobacteria	3086	34%
GCF_001488575.1	Halobacterium_hubeiense_JI20-1	Halobacteria	3143	38%
GCA_000018465.1	Nitrosopumilus_maritimus_SCM1	Thaumarchaeota	1795	40%
GCA_000336675.1	Halococcus_hamelinensis_100A6	Halobacteria	3400	37%
GCF_000685395.1	Candidatus_Nitrosotenuis_chungbukensis_MY2	Thaumarchaeota	1951	41%
GCF_002214165.1	Candidatus_Micrarchaeota_archaeon_Mia14	DPANN	960	54%
GCA_000496195.1	uncultured_archaeon_A07HB70_	unclassified	2513	37%
GCA_002737455.1	Thaumarchaeota_archaeon_	Thaumarchaeota	1365	34%
GCF_000328685.1	Natronococcus_occultus_SP4	Halobacteria	4035	35%
GCA_001595945.1	Thermoplasmatales_archaeon_SM1-50_	Thermoplasmata	1890	72%
GCF_002787055.1	Candidatus_Nitrosotenuis_sp_AQ6f_AQ6F	Thaumarchaeota	1912	37%
GCA_002737445.1	Candidatus_Nitrosoarchaeum_sp_	Thaumarchaeota	1389	33%
GCF_000337095.1	Halogeometricum_pallidum_JCM_14848	Halobacteria	4055	34%
GCA_001871475.1	Candidatus_Micrarchaeota_archaeon_CG1_02_47_40_	DPANN	1150	77%
GCF_000337395.1	Halorubrum_litoreum_JCM_13561	Halobacteria	2943	35%
GCF_000337675.1	Natronococcus_amylolyticus_DSM_10524	Halobacteria	4146	35%
GCA_000496175.1	uncultured_archaeon_A07HR67_	unclassified	2889	34%
GCF_000969905.1	Methanosarcina_vacuolata_Z-761	Methanomicrobia	3538	36%
GCF_000723845.1	Haloferax_alexandrinus_Arc-Hr	Halobacteria	3735	34%
GCF_001190965.1	Haloferax_gibbonsii_ARA6	Halobacteria	3744	33%
GCF_001469875.2	Haloferax_sp_SB3	Halobacteria	3719	34%
GCF_000337335.1	Halorubrum_distributum_JCM_10118	Halobacteria	3083	35%
GCF_000337035.1	Halorubrum_coriense_DSM_10284	Halobacteria	3312	37%
GCF_000336815.1	Haloferax_prahovense_DSM_18310	Halobacteria	3766	35%
GCA_000011005.1	Methanocella_paludicola_SANAE	Methanomicrobia	3004	51%
GCF_000337075.1	Halorubrum_hochstenium_ATCC_700873	Halobacteria	2850	34%
GCF_000723185.1	Thaumarchaeota_archaeon_N4	Thaumarchaeota	1854	40%
GCF_000025685.1	Haloferax_volcanii_DS2	Halobacteria	3857	33%
GCA_002762975.1	Candidatus_Diapherotrites_archaeon_CG11_big_fil_rev_8_21_1	DPANN	1219	65%
GCF_000337575.1	Natrialba_hulunbeirensis_JCM_10989	Halobacteria	3681	36%
GCF_001280425.1	Haloarcula_rubripromontorii_SL3	Halobacteria	3817	34%
GCF_000504565.1	Haloarcula_hispanica_N601	Halobacteria	3742	34%
GCF_000970045.1	Methanosarcina_sp_MTP4	Methanomicrobia	3422	40%
GCF_000955905.1	Candidatus_Nitrosotenuis_cloacae_SAT1	Thaumarchaeota	1797	40%
GCF_002844335.1	Haloarcula_taiwanensis_Taiwanensis	Halobacteria	3612	33%

GCF_000336955.1	Haloferax_larsenii_JCM_13917	Halobacteria	3535	36%
GCF_002788215.1	halophilic_archaeon_True-ADL	Halobacteria	3235	37%
GCF_000470655.1	Halorhabdus_tiamatea_SARL4B_type_strain_SARL4B	Halobacteria	2991	35%
GCA_002688315.1	Candidatus_Woesearchaeota_archaeon_	DPANN	1003	63%
GCF_000755245.1	Halococcus_sediminicola_CBA1101	Halobacteria	3559	34%
GCF_002286985.1	Halorubrum_sp_WN019	Halobacteria	3200	32%
GCF_001593955.1	Halalkalicoccus_paucihalophilus_DSM_24557	Halobacteria	3774	33%
GCF_000224475.1	halophilic_archaeon_DL31	Halobacteria	3370	36%
GCF_000336795.1	Haloferax_lucentense_DSM_14919	Halobacteria	3513	34%
GCF_000230955.2	Halobacterium_sp_DL1	Halobacteria	3180	35%
GCF_000755225.1	Halapricum_salinum_CBA1105	Halobacteria	3309	40%
GCA_002686355.1	Candidatus_Pacearchaeota_archaeon_	DPANN	494	66%
GCF_000495475.1	Candidatus_Halobonum_tyrrellensis_G22	Halobacteria	3293	34%
GCF_000739555.1	Halolamina_rubra_CBA1107	Halobacteria	2761	37%
GCF_002355655.1	Halorubrum_trapanicum_CBA1232	Halobacteria	2879	31%
GCA_002779065.1	Candidatus_Diapherotrites_archaeon_CG08_land_8_20_14_0_2(	DPANN	1017	64%
GCF_000337375.1	Halorubrum_lipolyticum_DSM_21995	Halobacteria	3138	35%
GCF_001485535.1	Halobacterium_sp_CBA1132	Halobacteria	3024	38%
GCA_002841105.1	Candidatus_Altiarchaeales_archaeon_HGW-Altiarchaeales-1_	Altiarchaeales	2007	67%
GCF_001368915.1	Haloferax_massiliensis_Arc-Hr	Halobacteria	3730	30%
GCF_000970265.1	Methanosarcina_lacustris_Z-7289	Methanomicrobia	3264	36%
GCF_000334895.1	Halococcus_agarilyticus_197A	Halobacteria	3226	34%
GCF_000204415.1	Methanothrix_soehngenii_GP6	Methanomicrobia	2899	36%
GCA_002839605.1	Methanomicrobiales_archaeon_HGW-Methanomicrobiales-3_	Methanomicrobia	2538	42%
GCF_000336995.1	Halorubrum_aidingense_JCM_13560	Halobacteria	2908	34%
GCF_000214725.1	Methanobacterium_paludis_SWAN1	Methanobacteria	2367	32%
GCF_000296615.1	Halorubrum_sp_T3	Halobacteria	2960	39%
GCF_000744455.1	Methanobacterium_sp_SMA-27	Methanobacteria	2415	37%
GCF_000979425.1	Methanosarcina_sp_2_H_T_1A_3	Methanomicrobia	3291	35%
GCF_000979475.1	Methanosarcina_sp_2_H_T_1A_8	Methanomicrobia	3296	35%
GCF_002252875.1	Halorubrum_ezzemoulense_Ec15	Halobacteria	3017	32%
GCF_000979455.1	Methanosarcina_sp_2_H_T_1A_6	Methanomicrobia	3295	35%
GCF_000746205.1	Halorubrum_sp_BV1	Halobacteria	2617	34%
GCF_000327485.1	Methanoregula_formicica_SMSP	Methanomicrobia	2789	35%
GCF_000063445.1	Methanocella_arvoryzae_MRE50	Methanomicrobia	3058	36%
GCF_000336975.1	Haloferax_sp_ATCC_BAA-644	Halobacteria	3437	32%
GCF_000336855.1	Haloferax_sp_ATCC_BAA-646	Halobacteria	3477	32%
GCA_002839705.1	Methanobacteriales_archaeon_HGW-Methanobacteriales-1_	Methanobacteria	2412	39%
GCF_000337795.1	Haloferax_denitrificans_ATCC_35960	Halobacteria	3660	33%
GCF_000336835.1	Haloferax_sp_ATCC_BAA-645	Halobacteria	3475	31%
GCF_000812185.1	Candidatus_Nitrosopelagicus_brevis_CN25	Thaumarchaeota	1412	35%
GCF_000023945.1	Halorhabdus_utahensis_DSM_12940	Halobacteria	2919	35%
GCF_000685155.1	Candidatus_Methanoperedens_nitroreducens_ANME-2d	Methanomicrobia	3254	47%
GCF_000069025.1	Halobacterium_salinarum_R1_DSM_671_R1	Halobacteria	2671	35%
GCF_000969965.1	Methanosarcina_sp_WWM596	Methanomicrobia	3377	36%
GCF_000979385.1	Methanosarcina_sp_2_H_A_1B_4	Methanomicrobia	3141	34%
GCF_002156705.1	Natrialbaceae_archaeon_JW/NM-HA_15	Halobacteria	3608	33%

GCF_001304615.1	Methanosarcina_flavescens_E03_2	Methanomicrobia	2652	35%
GCA_000145985.1	Ignisphaera_aggregans_DSM_17230	Desulfurococcales	1930	43%
GCA_002839675.1	Methanomicrobiales_archaeon_HGW-Methanomicrobiales-1_	Methanomicrobia	2456	40%
GCF_000147875.1	Methanolacinia_petrolearia_DSM_11571_	Methanomicrobia	2777	32%
GCF_000026045.1	Natronomonas_pharaonis_DSM_2160_Gabara	Halobacteria	2738	32%
GCA_001402855.1	Methanosarcina_sp_795_	Methanomicrobia	2416	35%
GCF_000591055.1	Natronomonas_moolapensis_8_8_11	Halobacteria	2733	34%
GCA_000016385.1	Pyrobaculum_arsenaticum_DSM_13514	Thermoproteales	2298	39%
GCA_002688035.1	Candidatus_Diapherotrites_archaeon_	DPANN	786	62%
GCF_000007065.1	Methanosarcina_mazei_Go1	Methanomicrobia	3347	34%
GCF_000970005.1	Methanosarcina_sp_WH1	Methanomicrobia	3206	35%
GCA_001587635.1	Arc_I_group_archaeon_ADurb1213_Bin02801_	Methanomicrobia	1585	33%
GCF_000148385.1	Vulcanisaeta_distributa_DSM_14429	Thermoproteales	2420	38%
GCA_002720095.1	Euryarchaeota_archaeon_	ucEuryarchaeota	1559	67%
GCF_002844195.1	Haloferacaceae_archaeon_SYSU_A9-0	Halobacteria	4243	34%
GCF_000190315.1	Vulcanisaeta_moutnovskia_768-28	Thermoproteales	2357	38%
GCA_002083985.1	Candidatus_Altiarchaeales_archaeon_A3_	Altiarchaeales	1305	74%
GCF_000744315.1	Methanosarcina_soligelidi_SMA-21	Methanomicrobia	3257	34%
GCF_000403645.1	Salinarchaeum_sp_Harcht-Bsk1	Halobacteria	2978	35%
GCA_002254565.1	Candidatus_Altiarchaeales_archaeon_ex4484_2_	Altiarchaeales	1481	58%
GCF_000308215.1	Methanomassiliicoccus_luminyensis_B10	Thermoplasmata	2555	49%
GCA_000495675.1	uncultured_Acidilobus_sp_MG_	Acidilobales	1787	36%
GCA_002255055.1	Candidatus_Aenigmarchaeota_archaeon_ex4484_14_	DPANN	880	62%
GCA_001587595.1	Arc_I_group_archaeon_ADurb1013_Bin02101_	Methanomicrobia	1591	33%
GCF_000015205.1	Pyrobaculum_islandicum_DSM_4184	Thermoproteales	1966	36%
GCF_001484195.1	Thermococcus_celericrescens_DSM_17994	Thermococci	2331	38%
GCF_000011205.1	Sulfolobus_tokodaii_str_7	Sulfolobales	2770	38%
GCF_000447865.2	haloarchaeon_3A1_DGR	Halobacteria	2714	43%
GCF_000191585.1	Methanobacterium_lacus_AL-21	Methanobacteria	2451	31%
GCF_001282785.1	Halolamina_sediminis_halo7	Halobacteria	2756	34%
GCA_001587605.1	Arc_I_group_archaeon_ADurb1113_Bin01801_	Methanomicrobia	1597	32%
GCA_001587695.1	Arc_I_group_archaeon_B15fssc0709_Meth_Bin003_	Methanomicrobia	1843	34%
GCA_000495695.1	uncultured_Acidilobus_sp_CIS_	Acidilobales	1671	37%
GCA_002898395.1	archaeon_HR02_	unclassified	1939	55%
GCF_002214545.1	Thermococcus_thioreducens_OGL-20P	Thermococci	2215	37%
GCF_001950595.1	Natronomonas_sp_CBA1134	Halobacteria	2620	33%
GCF_000969885.1	Methanosarcina_thermophila_TM-1	Methanomicrobia	2597	32%
GCA_000495715.1	uncultured_Acidilobus_sp_OSP8_	Acidilobales	1693	36%
GCF_000017945.1	Ignicoccus_hospitalis_KIN4/I	Desulfurococcales	1448	43%
GCF_000243255.1	Methanoplanus_limicola_DSM_2279	Methanomicrobia	2967	37%
GCF_000235565.1	Methanosaeta_harundinacea_6Ac	Methanomicrobia	2399	35%
GCA_000019805.1	Pyrobaculum_neutrophilum_V24Sta	Thermoproteales	1966	40%
GCF_001481685.1	Ignicoccus_islandicus_DSM_13165	Desulfurococcales	1478	56%
GCA_002785505.1	Candidatus_Altiarchaeum_sp_CG03_land_8_20_14_0_80_32_61	Altiarchaeales	1334	59%
GCF_002813655.1	Methanobacterium_sp_MO-MB1	Methanobacteria	2280	36%
GCF_000017625.1	Methanoregula_boonei_6A8	Methanomicrobia	2513	33%
GCF_000784355.1	Methanolacinia_paynteri_DSM_2545	Methanomicrobia	2664	36%

GCF_001560915.1	methanogenic_archaeon_ISO4-H5	Thermoplasmata	1795	42%
GCF_000966265.1	Palaeococcus_ferrophilus_DSM_13482	Thermococci	2246	36%
GCA_001587655.1	Arc_I_group_archaeon_B03fssc0709_Meth_Bin005_	Methanomicrobia	1802	33%
GCA_002791795.1	Candidatus_Altiarchaeum_sp_CG_4_10_14_0_8_um_filter_32_8	Altiarchaeales	1116	58%
GCF_000151205.2	Thermococcus_sp_AM4	Thermococci	2231	34%
GCF_000504205.1	Methanolobus_tindarius_DSM_2278	Methanomicrobia	2886	30%
GCF_001571385.1	Methanofollis_ethanolicus_HASU	Methanomicrobia	2554	38%
GCF_002355635.1	Halopenitus_persicus_CBA1233	Halobacteria	2855	30%
GCF_001548675.1	Methanobrevibacter_sp_YE315	Methanobacteria	2029	34%
GCA_000437055.1	Methanobrevibacter_smithii_CAG_186_	Methanobacteria	1706	39%
GCF_001663375.1	Caldivirga_sp_MU80	Thermoproteales	2161	48%
GCA_001873845.1	Candidatus_Altiarchaeum_sp_CG2_30_32_3053_	Altiarchaeales	1353	74%
GCF_001412615.1	Pyrodictium_delaneyi_Su06	Desulfurococcales	2013	53%
GCF_000025285.1	Archaeoglobus_profundus_DSM_5631	Archaeoglobi	1785	35%
GCF_000243315.1	Metallosphaera_yellowstonensis_MK1	Sulfolobales	2680	36%
GCF_002201915.1	Methanopyrus_sp_KOL6	Methanopyri	1504	46%
GCF_000404225.1	Candidatus_Methanomassiliicoccus_intestinalis_Issoire-Mx1	Thermoplasmata	1815	35%
GCA_002204705.1	Thermoplasmatales_archaeon_B_DKE_	Thermoplasmata	1950	62%
GCF_000265525.1	Thermococcus_cleftensis_CL1	Thermococci	2033	33%
GCF_001477655.1	Methanobrevibacter_millerae_SM9	Methanobacteria	2209	39%
GCF_000213215.1	Acidianus_hospitalis_W1	Sulfolobales	2332	36%
GCA_001587575.1	Arc_I_group_archaeon_BMIXfssc0709_Meth_Bin006_	Methanomicrobia	1730	32%
GCF_002287195.1	Methanosphaera_cuniculi_1R-7	Methanobacteria	1605	38%
GCF_000223395.1	Pyrolobus_fumarii_1A	Desulfurococcales	1906	45%
GCF_000024185.1	Methanobrevibacter_ruminantium_M1	Methanobacteria	2143	33%
GCF_002813675.1	Methanobacterium_sp_MZ-A1	Methanobacteria	2351	35%
GCF_002072215.1	Methanobrevibacter_arboriphilus_JCM_13429_DSM_1125_DH1	Methanobacteria	1887	38%
GCA_000015805.1	Pyrobaculum_calidifontis_JCM_11548	Thermoproteales	2149	39%
GCF_002813695.1	Methanobacterium_subterraneum_A8p	Methanobacteria	2355	35%
GCF_000009965.1	Thermococcus_kodakarensis_KOD1	Thermococci	2237	34%
GCA_000495735.1	uncultured_Acidilobus_sp_JCHS_	Acidilobales	1634	33%
GCA_002855745.1	Thermofilum_sp_NZ13_	Thermoproteales	1930	73%
GCF_000092465.1	Staphylothermus_hellenicus_DSM_12710	Desulfurococcales	1586	36%
GCF_001458655.1	Methanobacterium_formicicum_	Methanobacteria	2347	31%
GCF_000328665.1	Methanomethylovorans_hollandica_DSM_15978	Methanomicrobia	2525	31%
GCF_000769655.1	Thermococcus_eurythermalis_A501	Thermococci	2180	35%
GCF_000246985.2	Thermococcus_litoralis_DSM_5473	Thermococci	2306	35%
GCF_001647085.1	Thermococcus_piezophilus_CDGS	Thermococci	1856	34%
GCF_000022365.1	Thermococcus_gammatolerans_EJ3_DSM_15229	Thermococci	2117	32%
GCA_000437835.1	Methanoculleus_sp_CAG_1088_	Methanomicrobia	1625	34%
GCF_001305655.1	Halanaeroarchaeum_sulfurireducens_M27-SA2	Halobacteria	2194	35%
GCF_000015225.1	Thermofilum_pendens_Hrk_5	Thermoproteales	1866	40%
GCA_002839645.1	Methanomicrobiales_archaeon_HGW-Methanomicrobiales-2_	Methanomicrobia	2624	35%
GCA_002789105.1	Candidatus_Altiarchaeum_sp_CG_4_9_14_0_8_um_filter_32_20	Altiarchaeales	916	56%
GCA_001856825.1	Thermoplasmatales_archaeon_I-plasma_	Thermoplasmata	1696	77%
GCA_001800825.1	Euryarchaeota_archaeon_RBG_19FT_COMBO_69_17_	ucEuryarchaeota	1986	73%
GCF_000015145.1	Hyperthermus_butylicus_DSM_5456	Desulfurococcales	1676	44%

GCA_002457555.1	Marine_Group_II_euryarchaeote_MED-G37_	ucEuryarchaeota	1089	61%
GCF_000306725.1	Methanolobus_psychrophilus_R15	Methanomicrobia	2841	32%
GCF_001484685.1	Thermococcus_sp_2319x1	Thermococci	2017	33%
GCF_002214465.1	Thermococcus_barossii_SHCK-94	Thermococci	1996	30%
GCF_000013725.1	Methanococcoides_burtonii_DSM_6242	Methanomicrobia	2406	31%
GCF_000221185.1	Thermococcus_sp_4557	Thermococci	2085	31%
GCA_000022445.1	Sulfolobus_islandicus_M.16.4	Sulfolobales	2736	37%
GCF_000251105.1	Methanocella_conradii_HZ254	Methanomicrobia	2436	32%
GCF_001748385.1	Vulcanisaeta_thermophila_CBA1501	Thermoproteales	2039	35%
GCF_000015945.1	Staphylothermus_marinus_F1	Desulfurococcales	1598	35%
GCF_000015825.1	Methanoculleus_marisnigri_JR1	Methanomicrobia	2430	30%
GCF_000253055.1	Thermoproteus_tenax_Kra_1	Thermoproteales	1959	31%
GCF_000585495.1	Thermococcus_nautili_30-1	Thermococci	2132	34%
GCA_002728275.1	Candidatus_Heimdallarchaeota_archaeon_	Asgard	1355	64%
GCA_000565255.1	Sulfolobales_archaeon_AZ1_	Sulfolobales	1975	37%
GCA_001940665.1	Candidatus_Odinarchaeota_archaeon_LCB_4_	Asgard	1584	45%
GCA_002116695.1	Acidianus_manzaensis_YN-25	Sulfolobales	2644	40%
GCF_000193375.1	Thermoproteus_uzoniensis_768-20	Thermoproteales	2114	35%
GCF_001602375.1	Methanoculleus_horonobensis_T10	Methanomicrobia	2375	30%
GCA_002457155.1	Marine_Group_II_euryarchaeote_MED-G35_	ucEuryarchaeota	1035	60%
GCF_000304355.2	Methanoculleus_bourgensis_MS2_MS2T	Methanomicrobia	2538	32%
GCA_002254865.1	Archaeoglobales_archaeon_ex4484_92_	Archaeoglobi	3686	52%
GCF_002214505.1	Thermococcus_siculi_RG-20	Thermococci	2099	32%
GCF_000430905.1	Methanocorpusculum_bavaricum_DSM_4179	Methanomicrobia	1689	33%
GCF_000015765.1	Methanocorpusculum_labreanum_Z	Methanomicrobia	1799	33%
GCF_002197185.1	Thermococcus_sp_5-4	Thermococci	1961	29%
GCF_000021965.1	Methanosphaerula_palustris_E1-9c	Methanomicrobia	2696	30%
GCA_002898355.1	archaeon_HR01_	unclassified	1916	51%
GCF_000350305.1	Thermoplasmatales_archaeon_BRNA1	Thermoplasmata	1465	32%
GCF_001729385.1	Methanobrevibacter_sp_A27	Methanobacteria	1744	30%
GCF_001571405.1	Methanoculleus_thermophilus_CR-1	Methanomicrobia	2171	29%
GCF_000196655.1	Methanohalobium_evestigatum_Z-7303	Methanomicrobia	2267	30%
GCF_000632495.1	Candidatus_Acidianus_copahuensis_ALE1	Sulfolobales	2376	36%
GCF_001433455.1	Thermococcus_barophilus_CH5	Thermococci	2520	36%
GCA_002762655.1	Candidatus_Altiarchaeum_sp_CG12_big_fil_rev_8_21_14_0_65_	Altiarchaeales	1138	56%
GCF_000018305.1	Caldivirga_maquilingensis_IC-167	Thermoproteales	2005	34%
GCA_002457595.1	Marine_Group_II_euryarchaeote_MED-G36_	ucEuryarchaeota	828	59%
GCF_002214585.1	Thermococcus_profundus_DT_5432	Thermococci	2075	31%
GCF_000816105.1	Thermococcus_guaymasensis_DSM_11113	Thermococci	2029	33%
GCA_000011125.1	Aeropyrum_pernix_K1	Desulfurococcales	1700	49%
GCA_000591035.1	Aeropyrum_camini_SY1_JCM_12091	Desulfurococcales	1645	28%
GCF_001017125.1	Methanoculleus_sediminis_S3Fa	Methanomicrobia	2410	29%
GCF_000499765.1	Methanobacterium_sp_MB1_	Methanobacteria	1956	31%
GCF_000013445.1	Methanospirillum_hungatei_JF-1	Methanomicrobia	3294	34%
GCF_000800805.1	Candidatus_Methanoplasma_termitum_MpT1	Thermoplasmata	1383	36%
GCA_001742785.1	Candidatus_Altiarchaeales_archaeon_IMC4_	Altiarchaeales	1328	70%
GCF_000320505.1	Methanobrevibacter_boviskoreani_JH1	Methanobacteria	1706	31%

GCF_000025505.1	Ferroglobus_placidus_DSM_10642	Archaeoglobi	2479	33%
GCF_000235685.2	Methanolinea_tarda_NOBI-1	Methanomicrobia	2010	30%
GCF_001886955.1	Halodesulfurarchaeum_formicicum_HSR6	Halobacteria	2078	32%
GCF_000970325.1	Methanococcoides_methylutens_MM1	Methanomicrobia	2245	31%
GCF_002214565.1	Thermococcus_radiotolerans_EJ2	Thermococci	1984	29%
GCF_001719125.1	Sulfolobus_sp_A20	Sulfolobales	2593	31%
GCF_000993805.1	Thermofilum_uzonense_1807-2	Thermoproteales	1641	52%
GCA_001726015.1	Methanohalophilus_sp_2-GBenrich_	Methanomicrobia	1980	30%
GCF_000012285.1	Sulfolobus_acidocaldarius_DSM_639	Sulfolobales	2243	35%
GCA_000258425.1	Fervidicoccus_fontis_Kam940	Fervidicoccales	1385	34%
GCF_002153915.1	Methanonatronarchaeum_thermophilum_AMET1	Methanonatronarcl	1481	45%
GCF_000385565.1	Archaeoglobus_sulfaticallidus_PM70-1	Archaeoglobi	2180	34%
GCF_002214485.1	Thermococcus_pacificus_P-4	Thermococci	1868	29%
GCF_001592435.1	Thermococcus_peptonophilus_OG-1	Thermococci	1974	33%
GCF_000621965.1	Methanobrevibacter_wolinii_SH	Methanobacteria	1670	32%
GCF_000446015.1	Thermofilum_adornatus_	Thermoproteales	1825	51%
GCF_001266655.1	Metallosphaera_sedula_ARS50-1	Sulfolobales	2297	33%
GCF_900079115.1	Sulfolobus_solfataricus_P1	Sulfolobales	2944	33%
GCF_000789255.1	Geoglobus_acetivorans_SBH6	Archaeoglobi	2159	39%
GCF_000091665.1	Methanocaldococcus_jannaschii_DSM_2661	Methanococci	1762	37%
GCF_000430485.1	Thermococcus_sp_PK	Thermococci	2175	32%
GCF_000194625.1	Archaeoglobus_veneficus_SNP6	Archaeoglobi	2072	31%
GCF_002214365.1	Thermococcus_celer_Vu_13	Thermococci	1911	30%
GCF_000813245.1	Thermofilum_carboxyditrophus_1505	Thermoproteales	1849	51%
GCF_000018365.1	Thermococcus_onnurineus_NA1	Thermococci	1934	29%
GCF_001462395.1	Pyrodictium_occultum_PL-19	Desulfurococcales	1617	48%
GCF_002287215.1	Methanocorpusculum_parvum_XII	Methanomicrobia	1688	30%
GCF_000300255.2	Candidatus_Methanomethylophilus_alvus_Mx1201	Thermoplasmata	1589	35%
GCF_000275865.1	Methanofollis_liminatans_DSM_4140	Methanomicrobia	2396	30%
GCF_002214385.1	Thermococcus_gorgonarius_W-12	Thermococci	1774	28%
GCF_000024625.1	Methanocaldococcus_vulcanius_M7	Methanococci	1695	35%
GCA_000014945.1	Methanothrix_thermoacetophila_PT	Methanomicrobia	1696	28%
GCF_000517445.1	Thermococcus_paralvinellae_ES1	Thermococci	2054	30%
GCF_001563245.1	Methanobrevibacter_olleyae_YLM1	Methanobacteria	1778	33%
GCF_000025525.1	Methanocaldococcus_sp_FS406-22	Methanococci	1790	34%
GCF_001729965.1	Methanosphaera_sp_WGK6	Methanobacteria	1550	36%
GCF_000711215.1	Methanomicrobium_mobile_BP	Methanomicrobia	1617	34%
GCF_000008645.1	Methanothermobacter_thermautotrophicus_str_Delta_H	Methanobacteria	1756	30%
GCF_000012545.1	Methanosphaera_stadtmanae_DSM_3091	Methanobacteria	1518	32%
GCF_000204925.1	Metallosphaera_cuprina_Ar-4	Sulfolobales	1894	29%
GCF_000007305.1	Pyrococcus_furiosus_DSM_3638	Thermococci	1979	29%
GCA_002356395.1	Methanothermobacter_sp_EMTCatA1_	Methanobacteria	1814	49%
GCF_000179575.2	Methanothermococcus_okinawensis_IH1	Methanococci	1576	31%
GCF_000152265.2	Ferroplasma_acidarmanus_fer1	Thermoplasmata	1879	31%
GCF_000215995.1	Pyrococcus_yayanosii_CH1	Thermococci	1786	28%
GCA_001875345.1	Marine_Group_III_euryarchaeote_CG-Epi1_	ucEuryarchaeota	1061	72%
GCF_000006175.1	Methanococcus_voltae_A3	Methanococci	1688	32%

GCA_001800815.1	Euryarchaeota_archaeon_RBG_19FT_COMBO_56_21_	ucEuryarchaeota	1672	70%
GCA_001875465.1	Marine_Group_III_euryarchaeote_CG-Epi2_	ucEuryarchaeota	1115	71%
GCF_001577775.1	Pyrococcus_kukulkanii_NCB100	Thermococci	2064	32%
GCF_900012635.1	Thermococcus_chitonophagus_	Thermococci	2076	30%
GCF_000211475.1	Pyrococcus_sp_NA2	Thermococci	1924	31%
GCF_000725425.1	Palaeococcus_pacificus_DY20341	Thermococci	1950	29%
GCF_000828575.1	Methanothermobacter_sp_CaT2	Methanobacteria	1739	28%
GCF_000949015.1	Acidiplasma_sp_MBA-1	Thermoplasmata	1750	38%
GCF_000186365.1	Desulfurococcus_mucosus_DSM_2162	Desulfurococcales	1345	29%
GCF_000019605.1	Candidatus_Korarchaeum_cryptofilum_OPF8_	Korarchaeota	1645	33%
GCF_001317345.1	Thermococcus_sp_EP1	Thermococci	1909	30%
GCA_001683555.1	Methanohalophilus_sp_DAL1_	Methanomicrobia	1892	34%
GCF_000011105.1	Pyrococcus_horikoshii_OT3	Thermococci	1801	30%
GCF_000011585.1	Methanococcus_maripaludis_S2	Methanococci	1718	29%
GCA_001515185.1	Hadesarchaea_archaeon_DG-33_	Hadesarchaea	862	75%
GCF_002078355.1	Ferroplasma_acidiphilum_Y	Thermoplasmata	1764	34%
GCF_000092185.1	Thermosphaera_aggregans_DSM_11486	Desulfurococcales	1368	34%
GCF_000231015.2	Desulfurococcus_amylolyticus_DSM_16532	Desulfurococcales	1422	29%
GCF_000008665.1	Archaeoglobus_fulgidus_DSM_4304	Archaeoglobi	2369	32%
GCF_000264495.1	Thermogladius_calderae_1633	Desulfurococcales	1400	32%
GCA_001875425.1	Marine_Group_III_euryarchaeote_CG-Bathy1_	ucEuryarchaeota	950	69%
GCF_000739065.1	Methanocaldococcus_bathoardescens_JH146	Methanococci	1614	32%
GCF_000195935.2	Pyrococcus_abyssi_GE5_	Thermococci	1862	28%
GCF_000214415.1	Methanotorris_igneus_Kol_5	Methanococci	1751	30%
GCA_002899815.1	Candidatus_Korarchaeota_archaeon_	Korarchaeota	1564	51%
GCF_000144915.1	Acidilobus_saccharovorans_345-15	Acidilobales	1478	32%
GCF_001006045.1	Geoglobus_ahangari_234	Archaeoglobi	1985	35%
GCA_000270325.1	Candidatus_Caldiarchaeum_subterraneum_	Thaumarchaeota	1730	39%
GCF_000317795.1	Caldisphaera_lagunensis_DSM_15908	Acidilobales	1491	30%
GCF_000711905.1	Methermicoccus_shengliensis_DSM_18856	Methanomicrobia	1558	37%
GCA_001508185.1	Methanosarcinales_archeaon_56_1174_	Methanomicrobia	1462	28%
GCA_002490245.1	Candidatus_Bathyarchaeota_archaeon_B24-2_	Bathyarchaeota	1404	47%
GCA_001507975.1	Euryarchaeota_archaeon_55_53_	ucEuryarchaeota	1386	28%
GCF_000376965.1	Methanothermococcus_thermolithotrophicus_DSM_2095	Methanococci	1624	34%
GCA_001515215.1	Hadesarchaea_archaeon_YNP_N21_	Hadesarchaea	1238	76%
GCF_000023985.1	Methanocaldococcus_fervens_AG86	Methanococci	1554	31%
GCF_000092305.1	Methanocaldococcus_infernus_ME	Methanococci	1437	28%
GCA_001593935.1	Candidatus_Bathyarchaeota_archaeon_B26-2_	Bathyarchaeota	1630	39%
GCA_001593865.1	Candidatus_Bathyarchaeota_archaeon_B24_	Bathyarchaeota	1576	35%
GCF_000258515.1	Thermococcus_zilligii_AN1	Thermococci	1789	28%
GCF_000017165.1	Methanococcus_vannielii_SB	Methanococci	1679	28%
GCF_000145295.1	Methanothermobacter_marburgensis_str_Marburg	Methanobacteria	1701	27%
GCF_000022545.1	Thermococcus_sibiricus_MM_739	Thermococci	1913	27%
GCF_000195915.1	Thermoplasma_acidophilum_DSM_1728	Thermoplasmata	1521	27%
GCF_001889405.1	Methanohalophilus_halophilus_Z-7982	Methanomicrobia	1987	27%
GCF_000017185.1	Methanococcus_aeolicus_Nankai-3	Methanococci	1489	27%
GCF_000263735.1	Pyrococcus_sp_ST04	Thermococci	1789	27%

GCF_002813085.1	Methanobrevibacter_smithii_KB11	Methanobacteria	1675	27%
GCF_002973515.1	Methanohalophilus_euhalobius_DSM_10369	Methanomicrobia	1841	27%
GCF_000025665.1	Aciduliprofundum_boonei_T469	Thermoplasmata	1521	27%
GCF_000327505.1	Aciduliprofundum_sp_MAR08-339	Thermoplasmata	1491	27%
GCF_002214525.1	Thermococcus_sp_P6	Thermococci	1568	27%
GCF_000011185.1	Thermoplasma_volcanium_GSS1	Thermoplasmata	1545	26%
GCF_000404165.1	Methanobrevibacter_sp_AbM4	Methanobacteria	1675	26%
GCF_000371805.1	Methanocaldococcus_villosus_KIN24-T80	Methanococci	1346	25%
GCF_000217995.1	Methanosalsum_zhilinae_DSM_4017	Methanomicrobia	1955	25%
GCF_000008265.1	Picrophilus_torridus_DSM_9790	Thermoplasmata	1563	25%
GCF_000025865.1	Methanohalophilus_mahii_DSM_5219	Methanomicrobia	1955	25%
GCA_000387965.1	Candidatus_Nanobsidianus_stetteri_	DPANN	647	24%
GCA_001766815.1	Candidatus_Syntrophoarchaeum_caldarius_	Methanomicrobia	1787	24%
GCA_001914405.1	Candidatus_Methanohalarchaeum_thermophilum_	Methanonatronarcl	2167	24%
GCF_000166095.1	Methanothermus_fervidus_DSM_2088	Methanobacteria	1296	22%
GCF_002761295.1	Methanohalophilus_portucalensis_FDF-1T	Methanomicrobia	2040	22%
GCA_000008085.1	Nanoarchaeum_equitans_Kin4-M_	DPANN	536	0%

Fraction of dark matter after arCOG based annotation
25%
32%
24%
34%
29%
29%
34%
33%
30%
29%
33%
28%
29%
27%
26%
31%
22%
29%
25%
27%
23%
21%
21%
32%
35%
42%
42%
42%
40%
39%
37%
37%
36%
36%
35%
35%
35%

34%

34%

34%

33%

33%

33%

33%

33%

33%

33%

33%

33%

33%

32%

32%

32%

32%

32%

31%

31%

31%

31%

31%

31%

31%

31%

30%

30%

30%

30%

30%

30%

30%

30%

30%

30%

30%

30%

30%

29%

29%

29%

29%

29%

29%

29%

29%

28%
28%
28%
28%
28%
28%
28%
28%
28%
28%
27%
27%
27%
27%
27%
27%
27%
27%
27%
27%
27%
27%
27%
27%
27%
27%
27%
27%
27%
27%
27%
27%
27%
27%
27%
27%
27%
27%
27%
26%
26%
26%
26%
26%
26%
26%
26%

26%
26%
26%
26%
26%
26%
26%
26%
26%
26%
26%
26%
26%
26%
26%
26%
26%
26%
26%
26%
26%
26%
26%
26%
26%
26%
26%
26%
26%
26%
26%
26%
26%
26%
26%
25%
25%
25%
25%
25%
25%
25%
25%
25%
25%
25%
25%

25%
25%
25%
25%
25%
25%
25%
25%
25%
25%
25%
25%
25%
25%
25%
25%
25%
25%
25%
25%
25%
25%
25%
25%
25%
25%
25%
25%
25%
25%
25%
25%
24%
24%
24%
24%
24%
24%
24%
24%
24%
24%
24%
24%
24%
24%
24%

24%
24%
24%
24%
24%
24%
24%
24%
24%
24%
24%
24%
24%
24%
24%
24%
24%
24%
24%
24%
24%
23%
23%
23%
23%
23%
23%
23%
23%
23%
23%
23%
23%
23%
23%
23%
23%
23%
23%
23%
23%
23%
23%
23%
23%
23%
23%

23%
23%
23%
22%
22%
22%
22%
22%
22%
22%
22%
22%
22%
22%
22%
22%
22%
22%
22%
22%
22%
22%
22%
22%
22%
22%
22%
22%
22%
22%
22%
22%
22%
22%
22%
22%
22%
22%
21%
21%
21%
21%
21%
21%
21%
21%
21%

21%
21%
21%
21%
21%
21%
21%
21%
21%
21%
21%
21%
21%
21%
21%
21%
21%
21%
21%
21%
21%
21%
20%
20%
20%
20%
20%
20%
20%
20%
20%
20%
20%
20%
20%
20%
20%
20%
20%
20%
20%
20%
20%
20%
20%
20%
20%

20%
20%
19%
19%
19%
19%
19%
19%
19%
19%
19%
19%
19%
19%
19%
19%
19%
19%
19%
19%
19%
19%
19%
19%
19%
19%
19%
19%
18%
18%
18%
18%
18%
18%
18%
18%
18%
18%
18%
18%
18%
18%
18%
18%
18%
18%
18%

18%
18%
18%
18%
18%
18%
18%
18%
18%
18%
17%
17%
17%
17%
17%
17%
17%
17%
17%
17%
17%
17%
17%
17%
17%
17%
17%
17%
17%
17%
17%
17%
17%
17%
17%
17%
17%
17%
17%
17%
17%
17%
17%
16%
16%
16%
16%

16%
16%
16%
16%
16%
16%
16%
15%
15%
15%
15%
15%
15%
15%
15%
15%
15%
15%
15%
14%
14%
14%
14%
14%
14%
14%
14%
14%
13%
13%
13%
13%
13%
13%
13%
13%
12%
11%
8%
16%
15%
16%
15%
15%
15%
12%
15%

20%
15%
15%
16%
15%
15%
17%
11%
15%
14%
15%
21%
19%
24%
11%
17%
19%
